# Exploring the frontiers of therapeutic breadth of antifungal peptides: A new avenue in antifungal drugs

**DOI:** 10.1093/jimb/kuae018

**Published:** 2024-05-06

**Authors:** Ihtisham Ul Haq, Sajida Maryam, Divine Y Shyntum, Taj A Khan, Fan Li

**Affiliations:** Department of Physical Chemistry and Technology of Polymers, Silesian University of Technology, M. Strzody 9, 44-100 Gliwice, Poland; Joint Doctoral School, Silesian University of Technology, Akademicka 2A, 44-100 Gliwice, Poland; Programa de Pós-graduação em Inovação Tecnológica, Universidade Federal de Minas Gerais, Belo Horizonte 31270-901, MG, Brazil; Department of Physical Chemistry and Technology of Polymers, Silesian University of Technology, M. Strzody 9, 44-100 Gliwice, Poland; Joint Doctoral School, Silesian University of Technology, Akademicka 2A, 44-100 Gliwice, Poland; Biotechnology Centre, Silesian University of Technology, B. Krzywoustego 8, 44-100 Gliwice, Poland; Division of Infectious Diseases & Global Medicine, Department of Medicine, University of Florida, Gainesville, FL, USA; Institute of Pathology and Diagnostic Medicine, Khyber Medical University, Peshawar, Pakistan; School of Life Sciences, Peking University, Beijing 100871, People's Republic of China

**Keywords:** Antifungal peptides, Sources, Synthetic peptides, In vitro/in vivo activity, Antibiofilm activity, Mode of action

## Abstract

The growing prevalence of fungal infections alongside rising resistance to antifungal drugs poses a significant challenge to public health safety. At the close of the 2000s, major pharmaceutical firms began to scale back on antimicrobial research due to repeated setbacks and diminished economic gains, leaving only smaller companies and research labs to pursue new antifungal solutions. Among various natural sources explored for novel antifungal compounds, antifungal peptides (AFPs) emerge as particularly promising. Despite their potential, AFPs receive less focus than their antibacterial counterparts. These peptides have been sourced extensively from nature, including plants, animals, insects, and especially bacteria and fungi. Furthermore, with advancements in recombinant biotechnology and computational biology, AFPs can also be synthesized in lab settings, facilitating peptide production. AFPs are noted for their wide-ranging efficacy, in vitro and in vivo safety, and ability to combat biofilms. They are distinguished by their high specificity, minimal toxicity to cells, and reduced likelihood of resistance development. This review aims to comprehensively cover AFPs, including their sources—both natural and synthetic—their antifungal and biofilm-fighting capabilities in laboratory and real-world settings, their action mechanisms, and the current status of AFP research.

**One-Sentence Summary:**

This comprehensive review of AFPs will be helpful for further research in antifungal research.

## Graphical Abstract

**Graphical Abstract:**
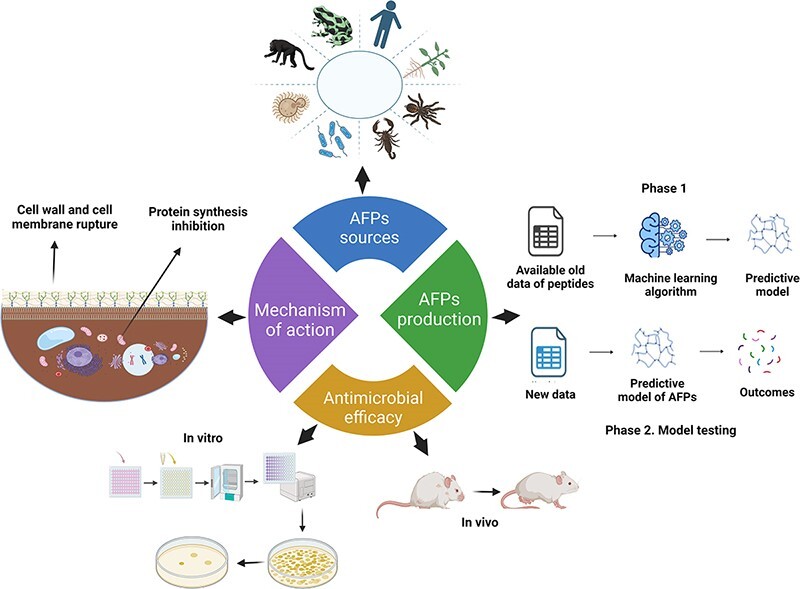

## Introduction

**Fig. 1.**
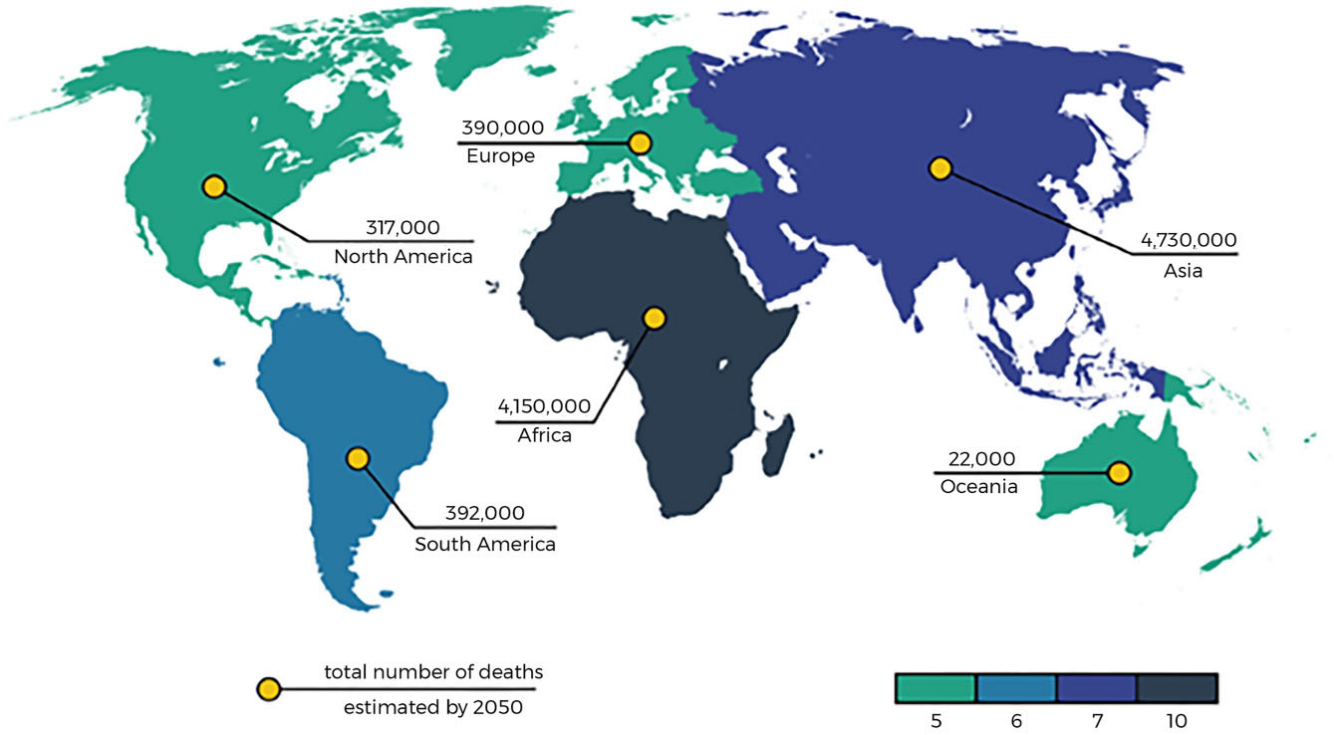
The estimated number of antimicrobial resistance-associated deaths worldwide in 2050 (Kamaruzzaman et al., 2019). Copyright (2019) MDPI.

The prevalence of invasive fungal infections has severely impacted public health, exacerbated by the limited options for antifungal treatments, which are further constrained by increasing drug resistance. This situation has significantly heightened the need for alternative antifungal solutions following the rise in infection rates and resistance to existing antimicrobials (Brower, 2018). Around 500 000 individuals are affected by antimicrobial resistance, and forecasts suggest that antimicrobial resistance could lead to 10 million fatalities over the next quarter-century, as depicted in Fig. 1 (Kamaruzzaman et al., 2019). Over the past 20 years, there has been a significant rise in antifungal drug resistance (Lionakis & Levitz, 2018). Around one-quarter of the global population has experienced fungal infections (Pfaller et al., 2019). There is a high number of antibacterial drugs available in the market. However, the range of antifungal drugs is limited. Only three classes of medications are available for systemic therapy such as polyenes (e.g. amphotericin B), triazoles (e.g. fluconazole, clotrimazole, miconazole, itraconazole, posaconazole, isavuconazole, voriconazole, and ketoconazole), and echinocandins (e.g. caspofungin and anidulafungin). Other drugs (e.g. 5-flucytosine, terbinafine, and griseofulvin) are available for adjunctive treatments.

Several pathogenic fungi such as *Candida, Cryptococcus, Fusarium, Paracoccidioides, Histoplasma, Rhizopus, Mucor, Pneumocystis, Scedosporium, Coccidioides*, and *Aspergillus* have been reported with wide-range multi-drug resistance (Gamaletsou et al., 2018; Huo et al., 2018). The design and development of antifungal drugs are complicated by the cellular resemblance to mammalian cells, including shared characteristics like a nuclear membrane (eukaryotic nature), analogous energy metabolism processes, and similar cytoskeleton organization (Mercer & O'Neil, 2020).

Historical data on antimicrobial development indicate that 78% of new antimicrobial agents were derived from natural origins, including plants, bacteria, animals, and mammals (McNair et al., 2018; Haq et al., 2019). Throughout the last several decades, these natural sources have continued to be a primary area of interest, undergoing regular investigation for the extraction of antifungal peptides (AFPs).

In 1948, *Bacillus subtilis* was found to produce antimicrobial peptides (AMPs) known as bacillomycin. Unlike its predecessors, bacillomycin showed limited antibacterial properties but was notably effective against fungal pathogens (Dahlman et al., 2021). The uncovering of bacillomycin in 1948 sparked scientific curiosity in the study of AFPs, leading researchers to delve deeper into AFPs that exhibit minimal cytotoxic and hemolytic effects (Fjell et al., 2011). In recent times, extensive research has been conducted on hundreds of Antifungal peptides (AFPs), revealing their wide-ranging potential against fungal infections and biofilm formation (Mahlapuu et al., 2020; Matejuk et al., 2010). As an illustration, by the year 2020, a total of 1133 AFPs had been documented in the Antimicrobial Peptide Database (https://aps.unmc.edu/database/anti) (Fernández de Ullivarri et al., 2020).

Nevertheless, efforts to discover additional AFPs are ongoing (Fry, 2018).

This paper aims to review the sources of AFPs comprehensively, address their efficacy in vitro and in vivo, their antibiofilm activities, and elucidate their mechanisms of action. Through this exploration, we seek to highlight the potential of AFPs as promising agents in the fight against fungal infections and their resistance, thereby contributing to developing more effective antifungal strategies.

## Fungal Infections: a Global Threat and Need for Antifungal Drugs

In recent years, fungal diseases have caused an estimated 1.6 million deaths annually worldwide, with more than one billion people suffering from severe fungal diseases. A wide range of human infections, from superficial to life-threatening systemic diseases, are reported to be caused by pathogenic fungi. These fungi significantly impact public health, with some species becoming particularly problematic due to their resistance to existing antifungal treatments (Oliveira et al., 2021). *Candida* spp.*, Aspergillus* spp.*, Cryptococcus* spp.*, Histoplasma capsulatum, Trichophyton, Microsporum, and Epidermophyton* spp. are widely reported fungal pathogens; their incidence and impact have evolved significantly over the years, reflecting a complex interplay of environmental, social, and medical factors (Rahim et al., 2017) (Du et al., 2021).This significant burden highlights fungi's threat to humans and the urgent need of novel antifungal medicines. The lack of compulsory public health surveillance for fungal diseases suggests that these estimates might be conservative, indicating a potentially more significant impact than currently recognized (Almeida et al., 2019). The impact of fungal diseases extends beyond health implications, encompassing economic burdens and challenges in public health management.

Furthermore, the COVID-19 pandemic has been associated with increased deaths from fungal infections, underscoring the dynamic and sometimes unexpected ways fungal diseases can affect public health (Gold et al., 2023). Moreover, fungi can form biofilms on medical devices and tissues resistant to most antifungal treatments (Krukiewicz et al., 2022). Biofilms protect fungal colonies from the immune response and antifungal agents, complicating the treatment of infections associated with catheters, prosthetic devices, and implants (Haq et al., 2023).

The antifungal pharmacopeia is much more limited than antibacterial agents, with only a few classes of antifungal drugs available. Fungal pathogens, similar to bacteria, have developed resistance mechanisms to current antifungal drugs. Resistance is particularly concerning with the widespread use of azoles in clinical and agricultural settings. Resistant strains, such as certain species of *Candida* (e.g. *Candida auris*), have emerged, making infections difficult to treat and leading to higher morbidity and mortality rates (Gow et al., 2022). AFPs are thought to be less prone to resistance due to their multifaceted mechanisms of action and ability to target fundamental aspects of fungal cell biology. Many antifungal drugs have significant toxicities and side effects. For example, amphotericin B, a polyene antifungal, can cause nephrotoxicity and infusion-related reactions. Azoles can induce liver toxicity and interact with a wide range of other medications, limiting their use in patients with complex medical conditions (Lewis, 2011). Some antifungal agents have a narrow spectrum of activity, making them effective against only a limited range of fungal pathogens. This specificity requires accurate identification of the pathogen, which may only sometimes be possible due to diagnostic limitations (Fisher et al., 2022). AFPs generally exhibit broad-spectrum activity against various fungi, including yeasts, molds, and some drug-resistant strains. Their broad-spectrum nature makes them potentially useful in treating infections where the pathogen is unknown (Marie & White, 2009). AFPs often have a broader range of mechanisms, including disrupting cell membranes, inhibiting cell wall synthesis, and interfering with intracellular targets. Some AFPs have multiple targets within the fungal cell, reducing the likelihood of resistance development (Li et al., 2022).

Developing new antifungal drugs is lengthy and costly, often taking over a decade from discovery to market. AFPs can be identified and synthesized relatively quickly using modern biotechnological methods. However, scaling up production and ensuring cost-effective manufacturing for clinical use remains a challenge (Dullius et al., 2020). Production processes are well-established for existing drugs. In summary, while conventional antifungal drugs have been the mainstay of fungal infection treatment, AFPs offer promising alternatives with potentially broader activity spectra, lower resistance rates, and reduced toxicity. Additionally, AFPs are rarely tested in biomedical sector particularly antimicrobial coatings compared with antibacterial coatings (Czerwińska-Główka et al., 2021) although AFPs can be goof alternative for coating especially food coatings since fungi causes food spoilage.

AFPs are less prone to drug resistance due to their rapid effects, pharmacodynamic properties, mechanisms of action such as efflux pumps, and regulation of signaling pathways (Kabra et al., 2019). Therefore, compared with other antifungal drugs, AFP-based therapy is less prone to resistance (Yeung et al., 2011). AMPs, such as lysozyme and histones are widely utilized against *Aspergillus fumigatus* hyphae and were not associated with an in-host acquisition of resistance. Nikkomycin Z, aureobasidin A, and VL-2397 are AFPs and have been evaluated for clinical trials (Rauseo et al., 2020).

## The Origins and Sources of AFPs

AFPs come from various sources, including microorganisms, plants, and animals. Bacteria and fungi produce peptides such as nisin and gramicidin. Plants contribute through components found in stems, seeds, and leaves, classified as thionins, defensins, and snakins. Additionally, marine environments are a rich source, with peptides like As-CATH4 and Myticusin-beta showing promising antifungal activities. Research also explores the potential of peptides derived from animals and chemically synthesized peptides for their antifungal properties. The discovery and isolation of AFPs have evolved. Typically, these discoveries involve identifying peptides with potential antifungal activity through screening natural sources or synthetic libraries, followed by isolation, characterization, and testing for antifungal properties against specific fungal pathogens (Zou et al., 2020).

### Mammals-derived AFPs and Their Efficacy

**Fig. 2.**
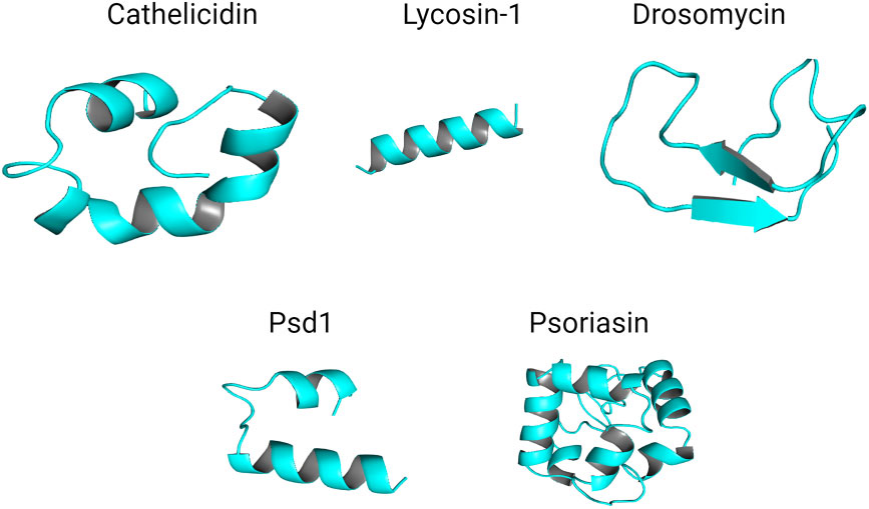
The three dimensional structures of AFPS such as Cathelicidin, Lycosin-1, Drosomycin, Psd1, and Psoriasin in cartoon form.

Mammals, including humans, are a valuable source of AFPs, which play an essential role in the innate immune system as a first line of defense against fungal infections. These peptides, which help protect the body against fungal pathogens that have a direct or inhibitory effect on growth, are part of the host's innate immunity and can be detected in different tissues and fluids. Histatins and defensins are two well-known mammal-derived AFP families (Bastos et al., 2018; Mahlapuu et al., 2020; Mbuayama et al., 2022). Histatins-1, 3, and 5 are members of the histatin family, among which the most potent AFP is histatin-5. Cell wall glycans, mainly β-1,3-glucan (Jang et al., 2010), and cell wall proteins, such as Ssa1 & Ssa2 of *Candida albicans*, are used by histatin-5 to bind the fungal cell wall and to enter the cell using their polyamine transporters Dur3 and Dur31 in an energy-dependent process (Puri & Edgerton, 2014). However, histatin-5 can also enter the cell through endocytosis or direct uptake via the interface with the plasma membrane (Moghaddam-Taaheri et al., 2021). Fungal cell viability and volume are lost due to the triggering of histatin-mediated release of K^+^ (Baev et al., 2002). The antifungal activity of histatins is also associated with adenosine triphosphate (ATP) efflux, inducing the formation of reactive oxygen species (ROS), inhibiting oxidative phosphorylation, and also chelating metal ions, which are essential cofactors for the metabolic activity of the fungal cell (Baev et al., 2004). Defensins and human neutrophil peptides (HNPs) are among animals’ most highly organized and stable AFPs (Polesello et al., 2017; Shafee et al., 2016). There are four HNPs, such as HNP-1, HNP-2, HNP-3, and HNP-4. HNPs kill fungi by depleting intracellular ATP (Martinez & Casadevall, 2006). Human epithelial cells produce four different defensin peptides, such as human β-defensin-1 (hBD-1), hBD-2, hBD-3, and hBD-4, that prevent fungal colonization of the skin, gastrointestinal tract, respiratory tract, and urogenital, tract. hBD-1 to -3 inhibit *C. albicans* hBD-2 inhibits *A. fumigatus* (Okamoto et al., 2004), and hBD-3 inhibits C*andida glabrata* (Inthanachai et al., 2021) by permeabilization of their membrane. Psoriasins, dermcidin, neuropeptides, melanocyte-stimulating hormones, and lactoferricins are other human-derived broad-spectrum AFPs (Mercer & O'Neil, 2013). The cathelicidins family contains diverse broad-spectrum AFPs mainly isolated from mammals (Oshiro et al., 2019). LL-37 and BMAP-28 are cathelicidins (Fig. 2) that exhibited anti-adhesive effects against clinically important *Candida* spp. isolated from vaginal infections (Durnaś et al., 2016).

### Plants: as a Potential Source of AFPs and Their Efficacy

**Fig. 3.**
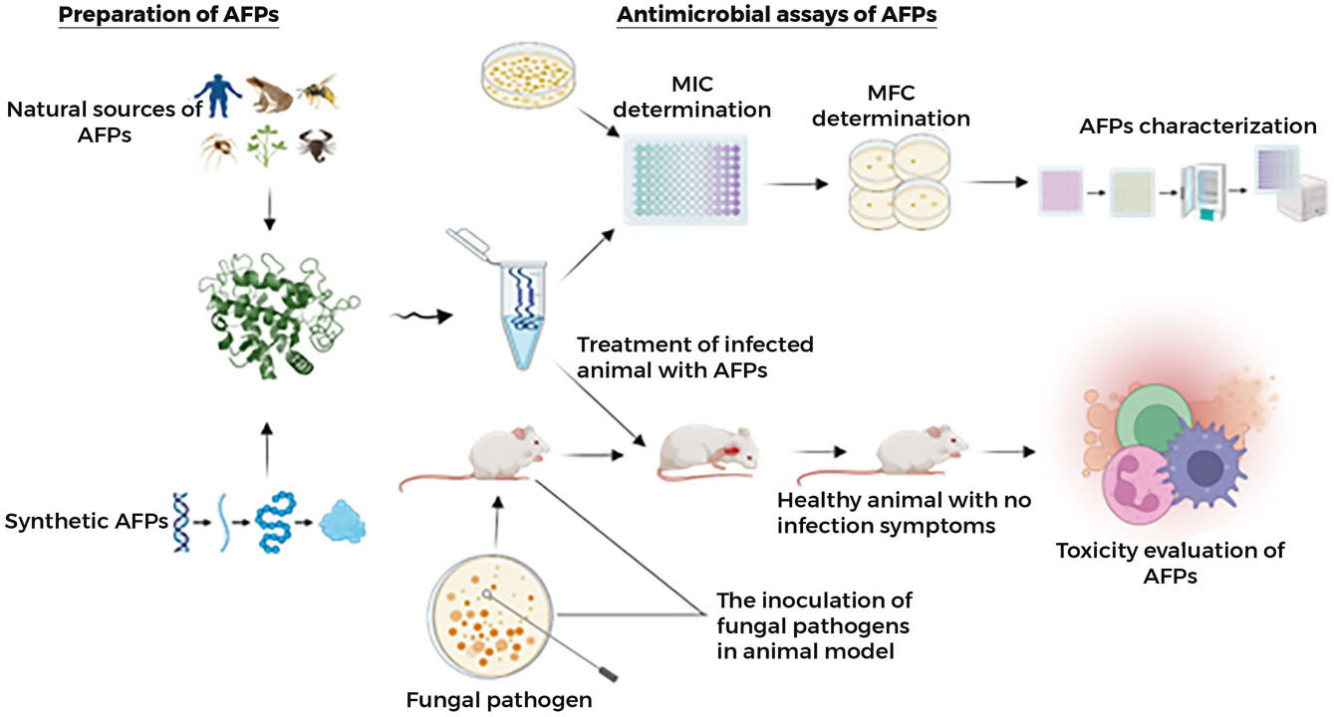
Schematic illustration of isolation/preparation of AFPs, antimicrobial activities of AFPs *in vitro* by Minimum Inhibitory Concentration/Minimum Fungicidal Concentration and *in vivo* by an animal. Created in Biorender.

Plants are a rich source of AFPs, essential to their innate immune system to fend off fungal pathogens. These peptides are part of a plant's defense mechanism, protecting against a wide range of fungal infections, and offer promising avenues for developing new antifungal agents with potential applications. Plant-derived defensins are cationic peptides found in the seeds, leaves, flowers, roots, and stems (Cools, Struyfs, Cammue, et al., 2017; Dos Santos et al., 2017; Fernández de Ullivarri et al., 2020; Skalska et al., 2020). Plants-derived AFPs inhibit fungi by interacting with the components of the cell wall and plasma membranes, such as β-glucans, chitin, and mannoproteins. Plants-derived defensins have been reported to target glycosphingolipid GlcCer in *Candida* spp. The peptides Psd1, ZmD32, and HsAFP1 are plant-derived AFPs with similar conserved regions. Psd1 causes disaggregation of the polysaccharide matrix of *Candida  alibicans* biofilms, and this peptide can attach to the fungal membranes, increasing cell roughness and decreased rigidity, leading to cell death (Gonçalves et al., 2017). Figure 3 is the schematic of the antimicrobial activity of the AFPs.

Balsamina seeds are found with AMPs (Ib-AMP1, Ib-AMP2, Ib-AMP3, and Ib-AMP4) that are highly enriched with cysteine derived from a single precursor protein (Vieira Bard et al., 2014) (Table 1). Ib-AMPs contain 20 amino acids, effective against *C. albicans and Aspergillus flavus*, and the disulfide bridges among amino acids are essential for activity (Thevissen et al., 2005).

**Table 1.** The Overview of Some Highly Active AFPs, Their Sources, and Minimum Inhibirory Concentration (MIC)


Peptides	Source	Fungal Pathogens	MIC	References

Histatin-5	Mammal	*Candida* spp.	1 µg–10 mg/mL	(Bastos et al., 2018)
hBD-3	Mammal	*C. glabrata*	50–75 µg	(Inthanachai et al., 2021)
ApoEdpL-W	Mammal	*C. glabrata*	10–25> µM	(Cutuli et al., 2000)
		*C. tropicalis*	2–5 µM	
		*C. albicans*	5–25> µM	
		*C. parapsilosis*	>25 µM	
LL-37	Mammal	*C. albicans*	2.5 µM–16 µg/mL	(Pinilla et al., 2022)
		*Vulvovaginal candidiasis clinical isolates*	2.5 µM–4 µg/mL	
Psoriasin	Mammal	*A. fumigatus, Malassezia furfur, Microsporum canis, Rhizopus oryzae, Saccharomyces cerevisiae, and Trichophyton mentagrophytes*	2 µM	(Hein et al., 2015)
BMAP-28	Mammal	*C. albicans & C. krusei*	2–32 µM	(Scarsini et al., 2015)
Psd1	Plant	*C. albicans*	20 Μm	(Gonçalves et al., 2017)
HsLin06_18	Plant	*C. albicans*	0.5 µM	(Cools et al., 2017)
ZmD32	Plant	*C. albicans, C. auris, C. glabrata, C. krusei, C. parapsilosis, C. tropicalis*	0.5–4.0 µM	(Kerenga et al., 2019)
Tn-AFP1	Plant	*C. tropicalis*	32 µg	(Mandal et al., 2011)
Vicilin-like peptides	Plant	*C. gloeosporioides*	200 µg	(Vieira et al., 2014)
PvD1	Plant	*Fusarium oxysporum, Fusarium solani, and Fusarium laterithium, Candida* spp.*, Kluyveromyces marxiannus, S. cerevisiae*	100 µg/ mL	(Skalska et al., 2020)
Iturin A	Bacteria	*Fusarium oxysporum f. sp. Niveum*	60 µg/mL	(Ji et al., 2020)
P-1	Bacteria	*Trichothecium roseum*	1 µg/mL	(Yan et al., 2018)
Syringostatin A & E	Bacteria	*A. fumigatus*	5–40 µg/mL	(Naing et al., 2015)
		*Mucor* spp.	6.25–100 mg/ mL	
OsAFP1	*Bacteria*	*Pleomorphomonas oryzae*	0.99 µg/mL	(Sagehashi et al., 2017)
		*Rhizoctonia solani*	1.48 µg/mL	
		*G. fujikuroi*	3.75 µg/mL	
Hyporientalin A	Fungi	*C. albicans.*	2.49–19.66 µM	(Touati et al., 2018)
VL-2397	Fungi	*Aspergillus* spp.*, C. neoformans, C. glabrata, Candida kefyr, T. asahii*	0.125→16	(Dietl et al., 2019)
Hafk	Fungi	*F. oxysporum* and *B. cinerea*	10 and 100 mM	Zhu et al., 2022)
NFAP and γ^NFAP^-opt	Fungi	*B. cinerea*	12.5–50 µg mL	(Tóth et al., 2020)
PAF, PAF^opt^ & Pγ^opt^	Fungi	*Fusarium* spp., *Aspergillus* spp., & *Cladosporium* spp.	1.56 –400 µg mL	(Tóth et al., 2020)
VLL-28	Insect	*Candida* spp.	12.5–50 µM.	(Notomista et al., 2015b)
AMP-17	Insect	*C. neoformans*	4–16 µg/mL	(Guo et al., 2017; Jiangfan et al., 2016)
Polybia-MPI	Insect	*Candida sppzhao*	8–64 µM	(Zhao et al., 2016)
Protonectin	Insect	*C. glabrata, C. albicans, C. parapsilosis, C. tropicalis, C. krusei*	8 µM–28 µM	(Mendes et al., 2004)
Lycosin-1	Insect	*Candida isolates*	8–128 µg/mL	(Tan et al., 2018)
TistH	Scorpion	*Candida* spp.*, A. flavus*	128 µg/mL)	(Machado et al., 2016)
ToAP2	Scorpion	*Sporothrix globosa*	156.25 µg/mL	(Buda De Cesare et al., 2020)
Ponericin-like AFP	Ant	*C. albicans*	0.0625 –10 µM	(Dodou et al., 2020)

### Microorganism-derived AFPs and Its Efficacy

Microorganisms are a prolific source of AFPs, offering a vast and diverse arsenal against fungal pathogens. Bacteria produce a variety of AFPs as part of their competitive strategies in various environments. Fungi themselves produce AFPs to compete against other fungal species for resources and space. These peptides can be particular to specific fungal pathogens (Lei et al., 2019; Ji et al., 2020; Zou et al., 2020). Fungal AFPs are echinocandins B peptide, which is thought of as a novel polypeptide-antibiotic isolated from *Aspergillus nidulans* var. *echinulatus. Bacillus* spp. has been acknowledged as a significant source of AFPs (Yan et al., 2018; Lei et al., 2019; Ji et al., 2020). Fungal-derived AFPs are lipopeptides and peptaibols produced as secondary metabolites. Leucinostatin A, an AFP antibiotic produced by *Penicillium lilacinum*, has been shown to possess antimicrobial properties against various *Candida* spp., including *C. albicans, Candida krusei, Candida tropicalis*, and *Candida guilliermondii* (Buda De Cesare et al., 2020). However, its potential for clinical use is limited due to its toxicity. Microorganisms-derived AFPs are discussed in the Table 1 in detail. Anti-biofilm activities of AFPs are mentioned in Table 2.

**Table 2.** The Anti-biofilm Potential of AFPs against Different Fungal pathogens

AFPs	Pathogen	Anti-biofilm activity	References
ApoEdpL-W	*C. glabrata*	ApoEdpL-W lacked efficacy against the mature stages of *C. glabrata* biofilms, although it demonstrated the ability to inhibit biofilm formation during the initial phases at a concentration of 200 µM.	(Rossignol et al., 2011)
Psd1	*C. albicans*	Psd1 showed antibiofilm effect against the biofilms of *C. albicans* at a concentration of 200 mM.	(Scarsini et al., 2015)
LL-37	*Candida*	LL-37 prevented *Candida* biofilm at a concentration of 2.5 µM to 64 µM.	(Pinilla et al., 2022)
BMAP-28	*C. albicans & C. krusei*	BMAP-28 inhibited 70%–90% biofilms of *C. albicans* and *C. krusei* isolates at a concentration16 µM.	Scarsini et al., 2015)
Tn-AFP1	*C. tropicalis*	Tn-AFP1 (*Trapa natans*) showed significant inhibitory effects against the biofilms of *C. tropicalis*.	(Inthanachai et al., 2021)
OSIP108	*C. albicans*	*C. albicans* form weak biofilms in presence of peptidevconcentrations ranging from 6.25 to 100 µM	(Delattin et al., 2014)
EntV	*C. albicans*	EntV is reported to reduce the 50% formed biofilms of *C. albicans*.	(Graham et al., 2017)
Fengycin	*C. albicans*	Fengycin remove 25%–100% of *C. albicans* biofilms grown on polystyrene plates.	(G. H. De Zoysa et al., 2018)
EntV68	*C. albicans*	EntV68 ruptured the biofilms of drug-resistant *C. albicans* on solid substrates.	(Graham et al., 2017)
Battacin	*C. albicans*	The linear battacin peptide exhibited inhibitory effects against the mature biofilms of *C. albicans*.	(Gayan Heruka De Zoysa et al., 2018)
AMP-17	*C. neoformans*	The peptide at concentration of 16∼32 µg/mL and 64∼128 µg/mL was able to constrain biofilm formation and eradicate the pre-biofilm.	(Tóth et al., 2020)
ZmD32	*C. albicans*	ZmD32 inhibited the biofilms with concentration of 20–50 µM.	(Kerenga et al., 2019)
Polybia-MPI	*C. glabrata*	Polybia-MPI inhibited the biofilms of *C. glabrata* on a polystyrene surface.	(Mendes et al., 2004)
Protonectin	*C. glabrata*	Protonectin disrupts the biofilms of *C. glabrata* by inducing the production of cellular ROS	(Mendes et al., 2004)
Lycosin-1	*C. albicans*	Lycosin-1 showed inhibitory effects with concentrations of 2.75–70.73 mM against fungal biofilms; however, 136.25–694.47 mM were able to rupture the mature biofilms.	(Ma et al., 2020)
Kaxins	*C. albicans, C. tropicalis C. glabrata*	Kaxins inhibited the biofilms of *C. albicans, C. tropicalis*, and *C. glabrata* with MIC ranges of 61.5– 246.1 mM.	(Brauner et al., 2018).
Killer peptide	*C. albicans*	MIC against early-stage biofilms was 124.2 mM, reducing more than 45% of the total mass.	(Paulone et al., 2017)
VLL-28	*Candida* spp.	VLL-28 effectively reduced the viability of the cells embedded in the mature biofilms, with MBEC_50_ and MBEC_80_ of 50 and 100 µM,	(Gläser et al., 2005)
HsLin06_18	*C. albicans*	HsLin06_18 strongly inhibited *C. albicans* biofilm formation with concentration of 0.4–0.7µM	(Cools et al., 2017)
IKIK2-NH2 and IRIK2-NH2	*C. albicans*	IKIK2-NH2, IRIK2-NH2, amphotericin B, and water eradicated the biofilms of *C. albicans* on the lense.	(Hong Wu et al., 2015)
Tn-AFP1	*C. tropicalis*	Tn-AFP1 inhibited 50% of biofilms at *C. tropicalis* 16 µg mL − 1	(Mandal et al., 2011)
Histatin 5	*C. albicans*	Histatin 5 reduces 50% of *C. albicans* biofilms at concentrations of 21 µg/mL.	(Curvello et al., 2019)

### Other Sources of AFPs and Their Efficacy

AFPs have been identified in various small animals, including wasps, scorpions, spiders, and insects. Notably, Antimicrobial Peptide-17 (AMP-17), derived from the transcriptome of Musca domestica when induced by *C. albicans*, exhibits significant antifungal capabilities (Guo et al., 2017; Jiangfan et al., 2016; Ma et al., 2020; Yang et al., 2022). Polybia-MPI, a peptide derived from the venom of the social wasp Polybia paulista, demonstrates strong fungicidal and antibiofilm properties, particularly effective against *C.* spp. at a concentration of 8–64 µM (Souza et al., 2005). The antifungal properties of Polybia-MPI, including its effectiveness, were also confirmed in a study by Wang et al. in 2014 (Wang et al., 2014). The Protonectin peptide, derived from the wasp Agelaia pallipes, is an AFP known for its antifungal solid efficacy against a variety of *Candida* spp., including *C. glabrata, C. albicans, Candida parapsilosis, C. tropicalis, and C. krusei* (Mendes et al., 2004). This peptide targets the fungal membrane, effectively disrupting *C. glabrata's* biofilms by triggering the production of cellular ROS (Wang et al., 2015). AFPs interrupt ROS balance, leading to oxidative damage to lipids, proteins, and DNA, resulting in fungal cell death (Cho & Lee, 2011). The venom from the Lycosa singoriensis spider has been found to contain AFPs like lycosin-1 (Fig. 2), which shows effectiveness against fluconazole-resistant strains of *C. albicans* (Faruck et al., 2016; Tan et al., 2018).

A diverse array of AFPs has been identified in scorpion venom (Buda De Cesare et al., 2020). The scorpion *Tityus stigmurus* produces venom that includes the α-helical peptide known as TistH (Richele JA Machado et al., 2016) its activity greatly vary from low to high against different fungal strains, having moderate activity against *C. albicans, C. tropicalis, Trichophyton rubrum*, and *A. flavus* (Richele JA Machado et al., 2016). Combining TistH with chitosan particles has been shown to enhance its antifungal effectiveness while maintaining its biocompatibility (Torres-Rêgo et al., 2019).

TistH, when tested in vivo on Swiss mice, exhibits minimal cytotoxicity and causes negligible inflammatory response (Richele JA Machado et al., 2016). The scorpions Tityus obscurus and Opisthacanthus produce potent AFPs, namely ToAP2 and NDBP57, which are both effective against *C. albicans* through the inhibition of fungi by membrane permeabilization, and disrupting cellular structure respectively (Buda De Cesare et al., 2020). Psoriasin peptide is potent AFP, have been identified in the skin lesions of psoriasis patients (Hein et al, 2015), and orthologs in amphibians (Matthijs et al., 2017) and in cattle (Regenhard et al., 2009) Psoriasin has been reported to be effective against drug-resistant dermatophytes such as *T. mentagrophytes, M. canis*, and *Epidermophyton floccosum* (Fritz et al., 2012). Although its mode of action remains largely unknown, previous studies have shown that psoriasin interferes with zinc homeostasis, and its sequestration could be a possible antimicrobial mechanism (Gläser et al., 2005). However, this peptide did not show antifungal activity against *C. albicans* but could bind -glucan and inhibit its adhesion to surfaces (Brauner et al., 2018). *Sulfolobus islandicus* produces the AMP VLL-28, representing the first archaeal kingdom-derived AFP (Notomista et al., 2015a). This cryptic peptide shows an effective antifungal activity against *C. albicans* and *C. parapsilosis* in terms of inhibition of growth and biofilms (Roscetto et al., 2018).

## Synthetic AFPs and Its Production

The constant emergence of antifungal drug resistance instincts the development of novel antifungal drugs. The design strategies for synthetic AFPs are usually inspired by membrane-active peptides, including host defense peptides and cell-penetrating peptides (Cheng et al., 2020; Norris et al., 2020; Skalska et al., 2020; Sharma et al., 2021; Struyfs et al., 2021; Jiang et al., 2022; Punginelli et al., 2022; Rodríguez-Castaño et al., 2023). Unlike naturally occurring AFPs, synthetic AFPs have better efficacies and comparatively less cytotoxic, less immunogenic, and low haemolytic activity (Pimienta et al., 2023). Duncan et al. (Duncan & O'Neil, 2013) synthesized an AFP named NP339 that showed broad-spectrum activity against several fungal pathogens and exhibited no cytotoxic or immunogenic activity. Kaxins are synthetic AFPs effective against several *Candida* spp., such as *C. albicans, C. tropicalis*, and *C. glabrata* strains (Burrows et al., 2006). Another synthetic decapeptide peptide, known as a killer peptide (KP), shows significant inhibitory effects against the planktonic cells of fluconazole-resistant *C. albicans* (Paulone et al., 2017). Additionally, transcriptional studies of *C. albicans* biofilms revealed the genes responsible for biofilm formation and hyphae development were found to be downregulated with AFP activity.

**Fig. 4.**
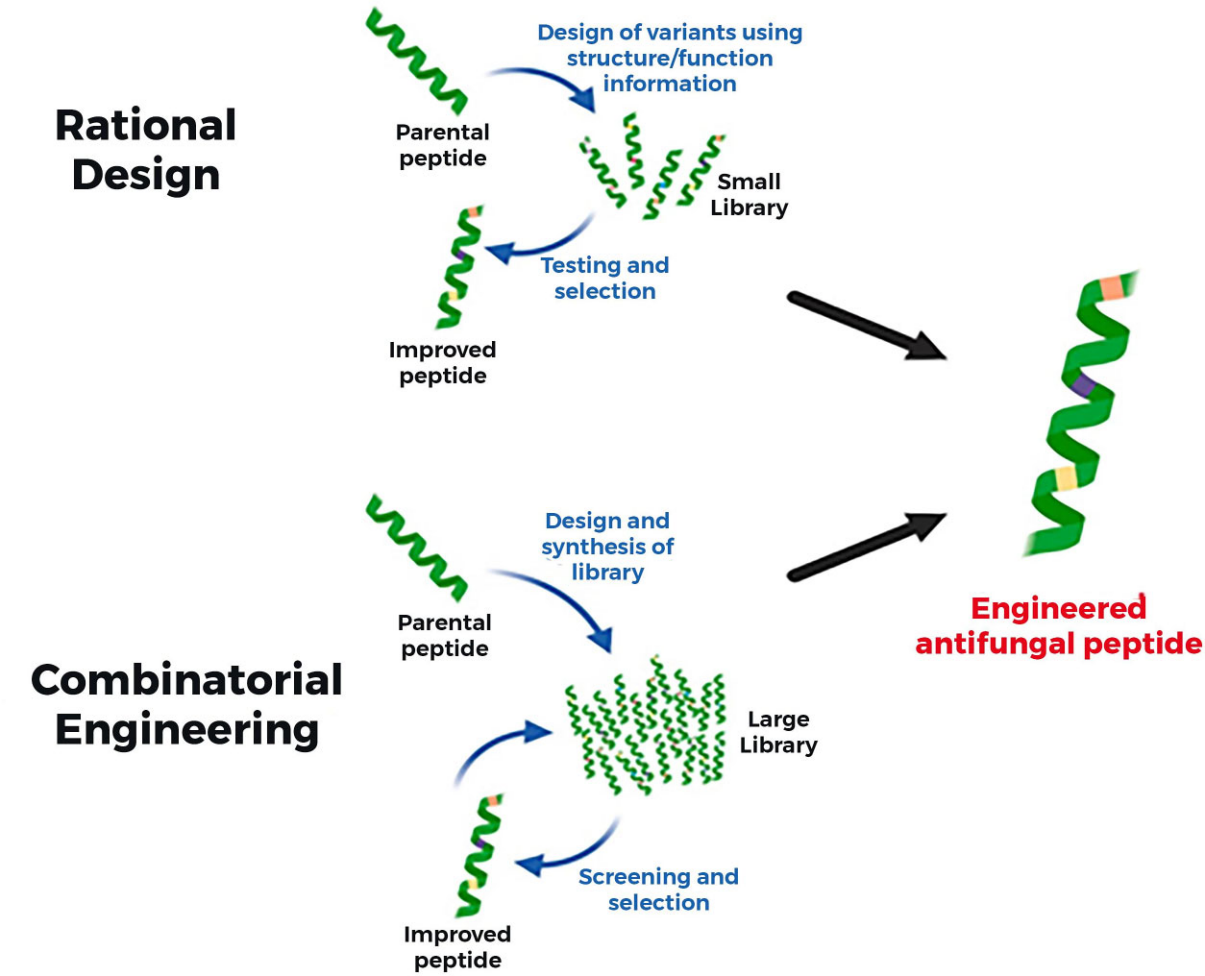
A depiction of two methodologies for designing antifungal peptides: **Rational Design** and **Combinatorial Engineering. Rational design** begins with a parental peptide, which is modified using structure/function information to create a small library of variants. These variants are then tested and selected based on their performance, leading to an improved peptide. **Combinatorial engineering** starts with a parental peptide but then involves designing and synthesizing a new, large library of variants that screen and select identify an improved peptide. These two approaches converge on the development of an engineered antifungal peptide, suggesting that the design process is iterative and can be refined through either rational design or combinatorial approaches to produce an optimized antifungal agent. Copyrights, 2023 Elsevier (Konakbayeva & Karlsson, 2023).

Identifying, characterizing, and optimizing novel antimicrobial agents is tremendously challenging using the currently available *in vitro* and *in vivo* testing approaches (Mercer et al., 2020). While some trials have been made toward the discovery and characterization of peptides, less attention has been paid to the optimization of these peptides. The same is true in part for clinical efficacy trials, which must be performed before any drug candidate can be approved for clinical use. AFP susceptibility testing also needs to be optimized to reduce overall delays in the discovery process (Mercer et al., 2020). Computational approaches can help understand the biology of peptides, which have added sufficient insights into peptide-based drug design (Lipkin & Lazaridis, 2017). For therapeutic applications, it is essential to determine the protease susceptibility, binding to membranes, and other macromolecule dose-exposure parameters. However, any deficiency can be compensated with different formulations, such as liposomal formulations (Vanzolini et al., 2022). The size of AFPs is comparatively tiny compared with proteins and can be optimized by different *silico* tools to improve biosynthesis (Agrawal et al., 2018). In this regard, electro-membrane filtration is one of the highly demanded technologies for synthesizing and purifying peptides (Wang & Ng, 2001). The current advances in computational approaches that can predict the drug target (Burkard et al., 2010; Porto et al., 2018), resistance development (Jena et al., 2014), and aid in the design of synthetic peptides (Blondelle & Lohner, 2000) as some example can be shown in Fig. 4.

AFPs not only inhibit the fungi but also denature mycotoxins, thus playing a central role in modern medicine and pharmaceutics (Avrahami & Shai, 2003; Zhang et al., 2020; Martínez-Culebras et al., 2021). Sharma et al. recently developed an in-silico model, “Deep-AFPpred,” that significantly identifies novel AFPs in protein sequences (Sharma et al., 2022). It includes the predicted peptides, their physicochemical characteristics, and their motifs. AFPs investigated in proteins can be chemically synthesized in a lab and experimentally verified for their antifungal action. Additionally, Ciociola et al. (Ciociola et al., 2023) used a bioinformatics approach and identified novel arginine and proline-rich candidacidal peptides. Computational tools such as AFP-MFL, a novel deep learning model, can accurately predict AFPs based on protein sequences without using structural information. AFP-MFL first constructs comprehensive feature profiles of AFPs, including contextual semantic information derived from a pre-trained protein language model, evolutionary information, and physicochemical properties that pave the way for the identification of novel AFPs (Xu et al., 2023). Machine learning algorithms, which have demonstrated excellent results in classifying biological data, are currently used as prospective data mining tools to create a precise prediction model for AFPs. Figure 4 shows the machine learning approach in predicting AFPs and other proteins. Several models can be used to predict the target of AFPs (Mousavizadegan & Mohabatkar, 2016). Chou's pseudo amino acid composition (PseAAC) has been used to translate peptide sequences into numeric values, and the SVM classifier performed for binomial classification of AFP (Carmen Rodríguez-Cerdeira et al., 2023). The accuracy of this PseAAC ++ SVM method is 94.76% and saves time and money in AFP screening and synthesis of novel peptides (C. Rodríguez-Cerdeira et al., 2023). New broad-spectrum AFPs with low haemolytic effects can be identified through bioinformatics filtering and peptidomic approach (Mousavizadegan & Mohabatkar, 2018). Singh et al. built a classifier that predicts AFPs with an accuracy and precision of 94%. Moreover, a temporal convolutional network-based binary classification method has been proposed by Singh et al. to discover new AFPs using the proteomic analysis of different organisms (Singh et al., 2022).

## *In Vivo* Efficacy of AFPs in Animal Models

The efficacy of various AFPs has been well established through numerous *in vitro* studies, demonstrating their potential as therapeutic agents in clinical medicine. However, the transition of AFPs into clinical trials and their practical application in healthcare is significantly hindered by the limited number of *in vivo* studies. This gap between laboratory research and real-world application has restricted the therapeutic use of AFPs. Despite these challenges, progress is being made with some newly identified and characterized AFPs undergoing *in vivo* studies. These advancements represent crucial steps towards understanding the potential clinical benefits of AFPs, laying the groundwork for their possible integration into medical treatments once their safety and efficacy are thoroughly validated through extensive *in vivo* research (Table 3). Different animal models, such as rabbits, primates, birds, guinea pigs, and most frequently murine models, have been used to test antifungal and antibiofilm peptides, as shown in Table 3. These models help evaluate the virulence of fungal pathogens. Disease progression can be easily monitored, and several biological variables can be controlled using these models. Non-vertebrate animals have also started to be utilized as animal models to optimize the screening of AFPs. The animal models are selected based on the mode of administration and therapeutic evaluation of drugs against fungal diseases. The method of administration of AFPs can be mucosal (oral and vaginal), superficial (skin and nails), gastrointestinal and lung, or systemic (intravenous, intraperitoneal) (H. Wu et al., 2015; G. H. De Zoysa et al., 2018).

**Table 3.** The *in vivo* efficacies of AFPs against different fungal infections

Peptide	Pathogens	Peptide activity	References
HsLin06_18	*C. albicans*	HsLin06_18exhibited significant antifungal activity against the biofilms of *C. albicans* on the urinary catheters in the rat's model and did not caused cytotoxicity.	(Vriens et al., 2016)
Drosomycin	*B. cinerea strain* B05-10*, C. gloeosporioides*	Drosomycin inhibited *B. cinerea strain* B05-10 and *Colletotrichum gloeosporioides* with 1.5 and 15 mM, respectively in *Caenorhabditis elegans* model.	(Xu et al., 2023)
Cathelicidins	*C. albicans*	Cathelicidins significantly reduce the biofilms of *C. albicans* in Murine model	(Aerts et al., 2008)
(IKIK)_2_-NH_2_ and (IRIK)_2_-NH_2_	*C. albicans*	Peptide treatment with amphotericin B reduces the keratitis infection and biofilms on the corneal surface of mice.	(K. Z. Hein et al., 2015)
EntV	*C. albicans, C. tropicalis, C. parapsilosis*, and *C. Glabrata*	EntV reduces epithelial invasion, inflammation, and fungal burden in a murine model having oropharyngeal candidiasis.	(Graham et al., 2017)
Psoriasin	*A. fumigatus* and *T. rubrum*	Psoriasin showed antifungal activity against *A. fumigatus* in a mouse model and *T. rubrum* skin infection in a guinea pig tinea pedis model.	(Dietl et al., 2019)
VL-2397	*A. fumigatus*	No *A. fumigatus* infection signs were observed in humans which were treated with peptide at concentration of 300, 600, and 1200 mg	(Mammen et al., 2019)
VL-2397	Antibiotic-resistant *C. glabrata*	This peptide reduces candidiasis caused by antibiotic-resistant *C. glabrata* in neutropenic mice.	(Wang & Ng, 2007)
sEntV^68^	*C. albicans*	sEntV^68^ significantly reduced fungal invasion in a mice that were treated with 100 nM	(Graham et al., 2017)

Several plant-derived AFPs proved effective in controlling fungal diseases and their biofilms in *vivo* models (Delattin et al., 2014). HsLin06_18 is an AFP derived from plant defensin HsAFP1 exhibited significant antifungal activity against the biofilms of *C. albicans* on the urinary catheters in the rat's model without causing cytotoxicity (Vriens et al., 2016). Drosomycin (Fig. 2) is an insect-derived defensin peptide reported to be active against fungi, such as *Botrytis cinerea* strain B05-10 and *Colletotrichum gloeosporioides*, respectively (Cohen et al., 2009). Combining OSIP108 peptide with Csf showed potent antifungal effects against *C. albicans in vivo* studies (Wang et al., 2014) using *Caenorhabditis elegans* (worm) as an animal model. *C. albicans* was inoculated in *C. elegans* and subsequently treated with the two combinations of OSIP108 and Csf, along with 0.6% DMSO as a negative control (Wang et al., 2014). The results showed that administration of OSIP108 without the Csf did not reduce infection symptoms in *C. elegans;* however, in combination, the worm survived and did not develop any symptom of infection. The authors suggested using OSIP108 in medical implants to reduce the burden of fungal biofilms on medical devices (Wang et al., 2014). At5 AFP showed promising results in treating wounds infected with *C. albicans* in mice and showed superiority over fluconazole for treating infection and accelerating wound healing (Xu et al., 2023).

Synthetic β-sheet peptides, specifically (IKIK)2-NH2 and (IRIK)2-NH2, have demonstrated efficacy *in vivo* studies targeting keratitis infections within a mouse model. These findings underscore the potential of these peptides as therapeutic agents in treating fungal keratitis, showcasing their ability to effectively manage this type of infection in animal models (H. Wu et al., 2015). A contact lens, previously inoculated with *C. albicans* to encourage biofilm formation, was then placed on the de-epithelialized cornea of mice. Within 18 hr, the mice exhibited classic symptoms of fungal keratitis infection, indicating successful establishment of the condition for further study (G. H. De Zoysa et al., 2018). Following the establishment of infection, the mice received topical treatments on the corneal surface, which included peptide solutions containing 3000 mg/L of each peptide, 1000 mg/L of amphotericin B, and water. The treatment led to a significant reduction in keratitis infection and completely eradicating the biofilms. In a separate study, *Enterococcus faecalis* was found to produce a broad-spectrum AFP named EntV, consisting of 68 amino acids. This peptide demonstrated effectiveness against various *Candida* spp., including *C. albicans, C. tropicalis, C. parapsilosis*, and *C. glabrata*.

Furthermore, it effectively reduced epithelial invasion, inflammation, and the fungal load in a murine model of oropharyngeal candidiasis (Graham et al., 2017). Patients with psoriasis were discovered to have the psoriasin peptide present in their skin lesions, a peptide with orthologues in amphibians and cattle, which exhibited antifungal activity. This activity was demonstrated *in vivo* through the successful treatment of *A. fumigatus* infections in a mouse lung model and *T. rubrum* skin infections in a guinea pig model of tinea pedis (Hein et al., 2015). An *in vivo* study demonstrated that the cyclic hexapeptide VL-2397 significantly reduced the burden of pulmonary aspergillosis in immunocompromised mice and extended their survival time compared to the control group (Dietl et al., 2019). Moreover, VL-2397 reduced candidiasis in neutropenic mice caused by antibiotic-resistant fungi *C. glabrata* (Wiederhold et al., 2020). Interestingly, VL-2397 also showed sufficient safety and tolerability in the first-in-human stage study (Phase I clinical trial) (Wang & Ng, 2007). The *in vivo* efficacies of AFPs are summarized in Table 3.

## Mechanism of Action of AFPs

The mechanisms of action of AFPs are varied and complex, involving multiple strategies to combat fungal pathogens. Many AFPs interact directly with the fungal cell membrane, leading to pore formation and disruption of membrane integrity. This causes leakage of vital intracellular contents, leading to cell death. Peptides may target specific membrane components, such as ergosterol, unique to fungal membranes, making this action highly specific and reducing the risk of harm to host cells (Dhandapani et al., 2022). Some AFPs target the fungal cell wall, a critical fungal survival and pathogenicity structure. These peptides can inhibit the synthesis of key cell wall components, such as β-glucans and chitin, weakening the cell wall and causing fungal cell lysis (Hasim & Coleman, 2019). Certain AFPs can inhibit the activity of essential fungal enzymes, disrupting critical physiological processes within the fungal cell. This includes enzymes involved in synthesizing the cell membrane or cell wall and those required for energy production and nutrient assimilation. The broad mechanisms of action of AFPs not only underline their potential as antifungal agents but also reduce the likelihood of developing resistance to fungal pathogens.

Different researchers reported different mechanisms of action; most of the peptides target chitin to kill fungal cells (Wang et al., 2019). AFPs mainly present their activity through membrane-associated mechanisms (Rautenbach et al., 2016). For instance, iturin causes oxidative stress by interacting with the target cell's cell membrane, leading to cell death. (Han et al., 2015). AFPs are also reported to kill or inhibit pathogenic fungi by preventing mycelium growth, spore germination, and deformation (Graham et al., 2017), while some AFPs affect the mycelium morphology only (Graham et al., 2017). The hyphae of *C.albicans* get distorted by the Bacillus AH-E-1 peptide, and the purified peptide stops the germination of spores, germ tubes, and the growth of hyphae (Chen et al., 2012). The EP-2 AFP produced by *B. subtilis* E1R-J can alter the fungal mycelium that leads to the inhibition of fungal growth (N. N. Wang et al., 2016).

Fernandez et al. highlight two key factors explaining mammals' lower toxicity of AFPs . First, the anionic nature of the fungal membrane, rich in phosphatidylinositol and phosphatidic acid, facilitates a stronger bond with the cationic charges of the peptide, unlike the predominantly neutral mammalian cell membranes, which contain phosphatidylcholine. Additionally, these peptides specifically target membrane lipids unique to fungi, leading to diminished toxicity in human hosts (Fernández de Ullivarri et al., 2020).

**Fig. 5.**
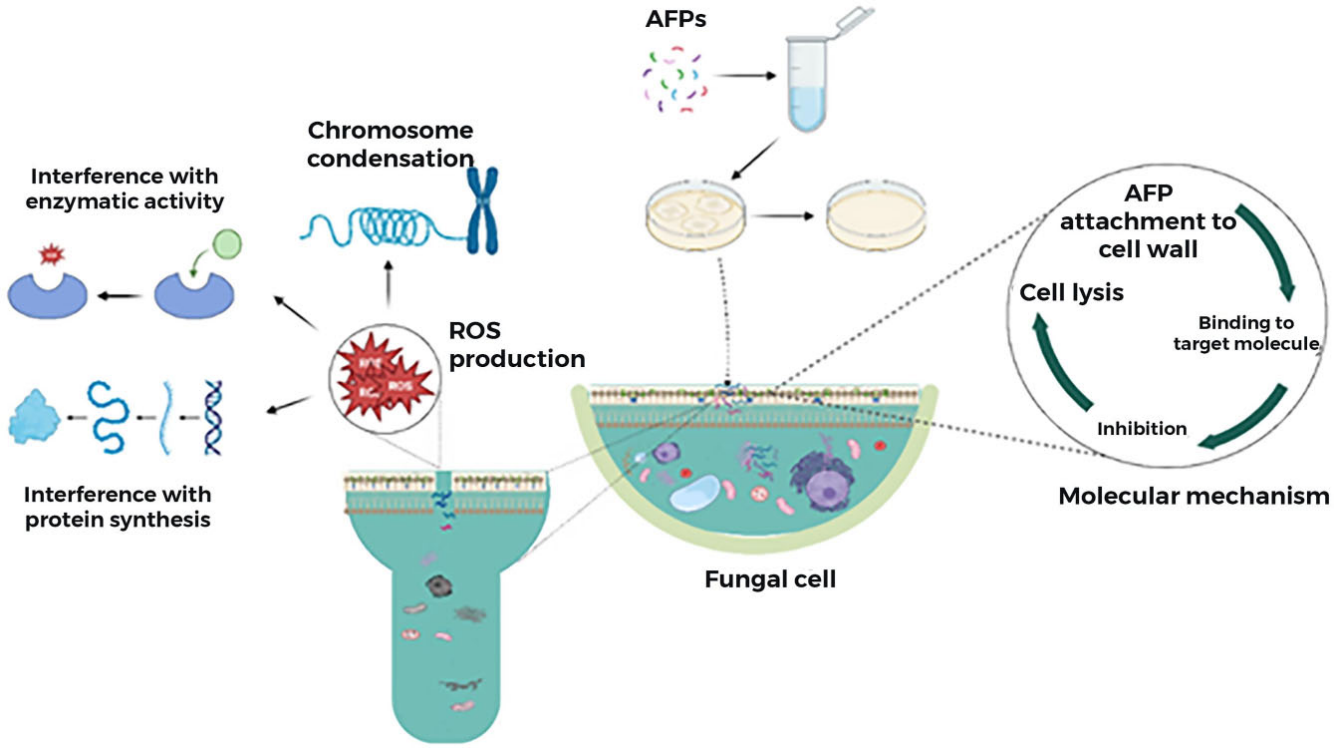
The attachment of the AFPs on fungal cell antimicrobial activity of. Mechanism of action; it shows the general mechanism of AFPs in fungal cell. Antifungal action of AFP: Its shows the different mode of antimicrobial action of AFPS i–e production of ROS, which interfere with fungal cellular processes, such as protein synthesis and enzymatic activity, and cause condensation of chromosomes. Figure created in Biorender.

Some AFPs target the fungal cell walls, usually composed of carbohydrates, glycoproteins, chitins, and other proteins (Neelabh et al., 2016; Garcia-Rubio et al., 2019; Figure 5). Studies confirmed that various AFPs influence the formation of these main components to damage the cell walls. Some peptides, such as the echinocandins, affect the synthesis of the fungal cell walls, which inhibit β-(1, 3)-glucan synthases. Caspofungin (Falagas et al., 2007), micafungin (Wasmann et al., 2018), and anifgin also show inhibitory effects similarly. Many antifungal substances extracted from *Streptomyces* species act on chitin. For example, Mizuhara et al. isolated cyclothiazomycin B1 from Streptomyces HA 125–40, which causes cell walls to rupture by interaction with the chitin, leading to the hydrolysis-mediated death of fungal cells (Mizuhara et al., 2011). The antifungal compound pradimicin (PRM) targets mannan, a fungi component cell wall. PRM binds to D-mannoside site on the cell walls of *C. albicans* forming a complex ‘pradimicin, D-mannoside, and calcium,’ which can destroy cell membrane integrity (Walsh & Giri, 1997). This mechanism of action has also been observed in *in vitro* studies against the various species of *Aspergillus, Candida*, and *Cryptococcus neoformans*. It does not cause significant human toxicity (Walsh & Giri, 1997). By accumulating ROS, PRM can induce apoptosis of *Saccharomyces cerevisiae* (Hiramoto et al., 2003). Other antifungal compounds, such as Benanomicin, have been reported to have a similar mechanism, like binding to cell-wall mannan (Yasuoka et al., 1995). The iturin lipopeptide produced by *Bacillus* also targets the fungal cell membranes (Maget-Dana & Peypoux, 1994). The fungal plasma membrane can be torn apart by actinomycin D, which breaks the membrane and causes the leakage of cellular contents (Zeng et al., 2019). Some AFPs, such as micafungin, are thought to alter metabolic pathways through protein synthesis inhibition and cell replication (Katragkou et al., 2017) 14-helical β-Peptides elicit toxicity against *C. albicans* by forming pores in the cell membrane and subsequently disrupting intracellular organelles (Lee et al., 2019). Cyclic lipopeptide AMP-jsa9 regulates the cellular protein level, targeting the cellular membrane structures and leakage of ions/proteins (Han et al., 2017). AFP, a KP, inhibits pathogens by producing oxidative stress and membrane permeabilization (Paulone et al., 2017).

## Advantages of AFPs

AFPs offer numerous benefits compared with existing antifungal medications, thanks to their distinctive modes of action and molecular targets. Their potential to recognize an extensive array of microbial targets also holds significant promise in averting the development of resistance (Rautenbach et al., 2016). The mechanisms of action of AFPs are diverse and include interactions with cell membranes, intracellular targets, and immunomodulation (Freitas & Felipe, 2023). Several AFPs specifically target conserved fungal molecules, such as glucosylceramide, mannosyldiinositol phosphorylceramide, and enzymes involved in β-glucan synthesis. Consequently, there is a lower chance of cytotoxicity toward mammalian cells and a higher level of pathogen selectivity (Rautenbach et al., 2016). The exhibition of less cytotoxicity (Matejuk et al., 2010) is also due to the fungal membrane, which is negatively charged due to the higher concentration of phosphatidylinositol. Phosphatidic acid interacts more strongly with the cationic charges of the peptides than do the cell membranes of mammals, which are primarily neutral to the host mammals because of the high concentration of phosphatidylcholine. Certain AFPs target membrane lipids specific to fungi absent from mammalian cells, decreasing toxicity (Nguyen et al., 2011; Rautenbach et al., 2016). These microbes target fungal membranes, various cell wall constituents, and substances involved in physiological processes, such as the synthesis of RNA, DNA, and proteins and the cell cycle (Van der Weerden et al., 2013; Bondaryk et al., 2017). Silico peptide optimization holds excellent potential for generating new peptides or optimizing naturally occurring ones (Porto et al., 2018). Numerous AFPs have been expressed transgenically in various crops, offering protection against fungal pathogens without apparent effects on plant health or crop yield (Li et al., 2011; Muramoto et al., 2012). In a lab setting, AFPs have also been demonstrated to regulate mammalian fungal infections (Kondori et al., 2011; Rossi et al., 2012). An increasing amount of reports about these AMPs’ possible uses in food (Thery et al., 2019). Peptides, whether natural or artificial, and their modified derivatives may serve as the building blocks for novel compounds effective against fungi (Ciociola et al., 2016). Additionally, AFPs can be very good alternative for antifungal and biodegradable coatings in food products (Janik et al., 2022).

## Challenges: what Next for AFPs

The challenges that slow down the commercialization of peptide-based antifungal drugs are specificity, safety, stability of the formulations, delivery strategies, and regulatory barriers. Costly processes that extract and purify peptides from natural processes and recombinant biotechnological approaches are recommended for higher yields (Lin & Ng, 2008). Instead, recombinant biotechnological approaches have enabled peptidic production, but they must still be stringently optimized to scale up procedures successfully. Chemical synthesis is only feasible for short peptides with great-value applications. According to the new documents, the criteria for clinical studies of antimicrobials need to be straightforward and increase the range of volunteers for clinical trials (Wang & Ng, 2001). Strategies such as rational design and combinatorial engineering can reduce AFP limitations and improve its physiochemical and biological characteristics (Garrigues et al., 2017; Konakbayeva & Karlsson, 2023; Tóth et al., 2022). Based on the chemical behavior of the AFPs, manufacturing costs, and high reported antifungal agents in literature, AFPs are one of the most commercially attractive candidates for drug discovery (V. M. Duncan & D. A. O'Neil, 2013). Regulatory bodies, researchers/scientists, and biotechnological and pharmaceutical companies must collaborate to set an integral plan to enhance the thrust toward developing peptide-based antifungal therapies. The broad-spectrum inhibitory potential of peptides against fungal planktonic cells and biofilms, together with safety profile and stability to high temperatures, altered pH, and proteolysis, represent a solid basis for the development of AFPs as antimicrobial therapeutic agents for clinical use (Scarsini et al., 2015). Additionally, the wide breadth of some antifungals and their antibacterial properties is predominantly inspiring, thus representing their potential efficacy against polymicrobial infections (Perez-Rodriguez et al., 2022; Makambi et al., 2023; Shaban et al., 2023).

Furthermore, AFPs need to be stabilized, more soluble, and remain active for an extended period in complex matrices like food (Delgado et al., 2016; Thery et al., 2019). Low peptide concentrations and the synthesis and purification of antifungal AMPs from natural sources also limit their use (Marcos López et al., 2020). For the large-scale production of AFPs, chemical synthesis is still too costly and not always practical (Marcos López et al., 2020). A peptide taken orally is susceptible to proteolytic digestion by pepsin and trypsin, two digestive tract enzymes. Moreover, systemic administration may cause brief half-lives in vivo, protease degradation, and blood cytotoxic profiles (Kumar et al., 2018). Various factors, including pH, temperature, metal ions, UV light, and the activity of multiple proteases, can impact peptide stability. It has been discovered that peptides can function in pH environments that are both acidic and alkaline, demonstrating that they are not always dependent on neutral pH levels (Xin Zhao et al., 2013). In addition, specific AFPs’ activity is influenced by a few additional ions, including K+, Na+, Mg 2+, and Ca 2+ (N. Wang et al., 2016). Different phospholipids and sphingolipids are targeted explicitly by several peptides that have been found to interact specifically with the fungal cell wall or membrane. Target interaction determines whether peptides internalize or remain outside the fungal cell. Regardless of their possible absorption, AFPs can affect intracellular targets. ROS, autophagy, vacuolar dysfunction, disturbance of cation homeostasis, mitochondrial dysfunction, disruption of the cell cycle, and ATP efflux are just a few of the actions that may result from this (Struyfs et al., 2021). Natural source purification is insufficient to produce sufficient amounts of AMP, and large-scale AMP chemical synthesis is more expensive than small-molecule drug production (Koo & Seo,^ 2019^; de Ullivarri et al., 2020).

Nevertheless, chemical optimization and novel delivery techniques may overcome the undesirable characteristics preventing the use of peptides (Neelabh et al., 2016). AFPs help treat fungal infections, but there is still potential for development in a few areas. These include hemolytic activity, low selectivity, toxicity, and instability, especially in naturally occurring peptides, brought on by host enzyme degradation (Matsuzaki,^ 2019^; Lima et al., 2021).

## Conclusion and Future Remarks

AFPs have captivated the interest of researchers worldwide due to their broad-spectrum antifungal capabilities and the low likelihood of inducing drug resistance. There has been a gradual yet notable advancement in the development of AFPs, attributed to their minimal cytotoxicity and hemolytic activity, positioning them as ideal candidates for enriching the arsenal of antifungal drug classes. On a laboratory scale, AFPs have demonstrated significant therapeutic potential *in vivo* studies. Yet, there is a critical need for extensive experimental research to comprehensively understand their biological attributes and facilitate their progression to clinical trials. It has been over 15 years since the last introduction of antifungal classes, such as echinocandins and pneumocandins, into the market, underscoring the limited variety of available antifungal drugs. Unfortunately, since the 2000s, major pharmaceutical companies have shown minimal interest in advancing antifungal research, leaving the field primarily to university laboratories and research institutions. These entities have been at the forefront of exploring AFPs, showcasing their potential as effective agents against fungal infections through diligent study and experimentation.

Advances in rational design and combinatorial engineering promise to overcome existing limitations and enhance the physicochemical and biological properties of AFPs. Given the prolonged timeline required for developing new antimicrobials, leveraging our knowledge of AFPs is essential for the innovation of novel antifungal therapies. Regulatory bodies, researchers, and biotech/pharmaceutical companies must collaborate to establish a comprehensive strategy to accelerate the development of AFP-based antimicrobial treatments. This cooperative approach is crucial for addressing the urgent need for new antifungal agents and mitigating the impact of fungal diseases.
